# Transforaminal approach versus interlaminar approach: A meta-analysis of operative complication of percutaneous endoscopic lumbar discectomy

**DOI:** 10.1097/MD.0000000000020709

**Published:** 2020-06-19

**Authors:** Jianjian Yin, Yuqing Jiang, Luming Nong

**Affiliations:** Department of Orthopedics, The Affiliated Changzhou No.2 People's Hospital with Nanjing Medical University, Changzhou, China.

**Keywords:** lumbar disc herniation, meta-analysis, operative complication, percutaneous interlaminar endoscopic discectomy, percutaneous transforaminal endoscopic discectomy

## Abstract

**Background:**

To systematically analyze the differences of complications between percutaneous transforaminal endoscopic discectomy (PTED) and percutaneous interlaminar endoscopic discectomy (PIED) in the treatment of lumbar disc herniation.

**Methods:**

We performed a systematic search in MEDLINE, EMBASE, PubMed, Web of Science, Cochrane databases, Chinese Biomedical Literature Database, CNKI, and Wanfang Data for all relevant studies. All statistical analysis was performed using Review Manager Version 5.3.

**Results:**

A total of 15 articles with 1156 study subjects were included, with 550 patients in PTED group and 606 patients in PIED group. The results of the meta-analysis showed that postoperative dysesthesia (odds ratio [OR] = 0.61, 95% confidence interval [CI], 0.33–1.13), nerve root injury (OR = 1.22, 95% CI, 0.30–5.02), surgical site wound complications (OR = 1.26, 95% CI, 0.29–5.40), recurrence (OR = 1.09, 95% CI, 0.54–2.21), conversion to open surgery (OR = 1.26, 95% CI, 0.33–4.81), incomplete decompression (OR = 1.62, 95% CI, 0.43–6.09), and total complication (OR = 0.72, 95% CI, 0.49–1.06) showed no significant differences between the PTED group and the PIED group, while the PTED group had significantly better results in dural tear compared with the PIED group (OR = 0.31, 95% CI, 0.13–0.79).

**Conclusions:**

Dural tear was significantly less occured in PTED compared with PIED. The postoperative dysesthesia, nerve root injury, surgical site wound complications, recurrence, conversion to open surgery, incomplete decompression, and total complication did not differ significantly between PTED and PIED in the treatment of lumbar disc herniation.

## 1 Introduction

Since the first complete removal of the nucleus pulposus of lumbar disc herniation (LDH) by Mixter and Barr^[1]^ in 1934, the operation has been an effective method for the treatment of LDH. Owing to the advances in technology of minimally invasive spine surgery (MISS), percutaneous endoscopic lumbar discectomy (PELD) has routinely performed in recent years for LDH. Transforaminal (TF) and interlaminar (IL) are 2 major approaches of MISS with different characteristics and indications according to the surgical approaches clinically. Multiple studies^[2–5]^ have attested to the smaller incision, less tissue damage, effective in surgical outcomes, and long-term prognosis outcome of PELD via TF approach. For the cases with narrow foramen and high iliac crest, especially in L5/S1, the IL approach can be used properly.^[6,7]^ However, limited by the narrow operating space and field of vision, as well as the steep learning curve and other technical characteristics, there are also frequent reports^[8–10]^ of serious complications during or after the treatment of LDH. At present, there is no systematic review and evaluation report on the incidence and characteristics of complications of percutaneous transforaminal endoscopic discectomy (PTED) and percutaneous interlaminar endoscopic discectomy (PIED) for LDH. Therefore, the purpose of our meta-analysis is to systematically analyze the differences of complications between PTED and PIED in the treatment of LDH, so as to provide evidence-based basis for clinical decision-making and prediction.

## 2 Materials and methods

### 2.1 Study selection and search strategy

A comprehensive search was performed in MEDLINE, EMBASE, PubMed, Web of Science, Cochrane databases Chinese Biomedical Literature Database, CNKI, and Wanfang Data databases to identify all relevant studies available from their inception to December 31th 2019. We also searched trial registries of ongoing trials. When the criteria for inclusion or exclusion of a study were controversial, the corresponding author was consulted. The search strategy followed the identification and screening guidelines established by PRISMA statement.^[11]^ The following Mesh search headings and key words were used: (“percutaneous transforaminal endoscopic discectomy, PTED, TF-PELD, percutaneous interlaminar endoscopic discectomy, PIED, IF-PELD, operative complication”). These terms were used in different Boolean combinations. We retrieved all eligible studies and evaluated the reference lists of the identified studies and reviews.

### 2.2 Inclusion criteria

We included the following studies from the meta-analysis:

(1) study design: comparing PTED with PIED for treatment of LDH patients,
(2) include more than 10 patients in each group (minimum of 12 patients),
(3) the studies provided surgical complication outcomes, and
(4) available data for each surgical regimen.

The most recent was used if dual (or multiple) studies were reported by the same institution. Study designs included randomized controlled trials (RCTs) and retrospective/prospective cohort or case-control studies.

### 2.3 Exclusion criteria

Studies were excluded if they met the following criteria:

(1) Studies that included patients suffering from spinal infection, acute fracture, tumor, deformity, osteoporosis, or rheumatoid arthritis.
(2) Duplicate studies; review articles; case reports; biomechanical and cadaveric studies.
(3) Studies involving more than 1 level segmental intervertebral disc herniation and re-operations.

### 2.4 Data extraction

Two reviewers independently extracted the relevant data from the reports. The extracted data described the characteristics of the investigations regarding study design, mean age, sample size, and follow-up period. The outcomes pooled in this analysis included postoperative dysesthesia, nerve root injury, dural tear, surgical site wound complications, recurrence, conversion to open surgery, incomplete decompression, and total complication. Disagreements were resolved by a third referee.

### 2.5 Risk of bias assessment

The randomized controlled studies were assessed by the Cochrane Back Review Group ^[12]^ (Table 1). If studies met at least 6 of the 11 criteria, the study was regarded as low risk of bias, otherwise the study was labeled as high risk of bias. The risk of bias of the cohort studies was assessed using the Newcastle–Ottawa scale.^[13]^ (Table 2). A maximum of 9 points allocated for quality of selection (4 points), comparability (2 points), exposure (3 points), or outcome of study participants (3 points). If studies met at least 5 points out of the 9 criteria, the study was considered to have low RoB. Conversely, the study was labeled as high RoB with only 4 or less met the 9 criteria. Risk of bias of the included studies were independently assessed by 2 review authors. Disagreement was resolved by consensus in all authors.

**Table 1:**
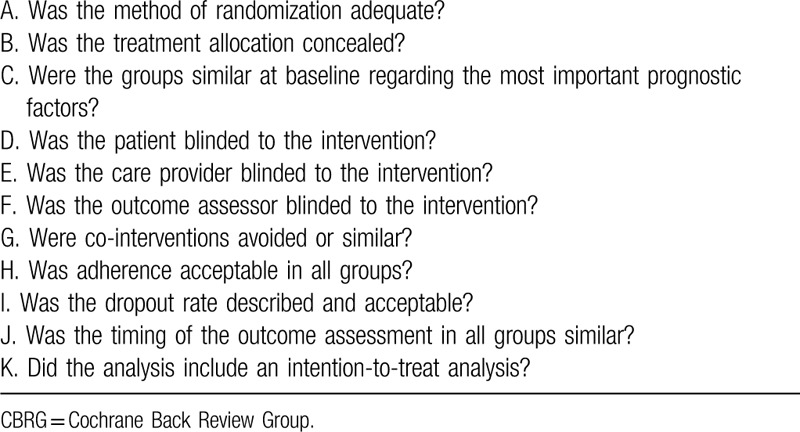
Risk of bias assessment of the randomized studies by the Cochrane Back Review Group (CBRG).

**Table 2:**
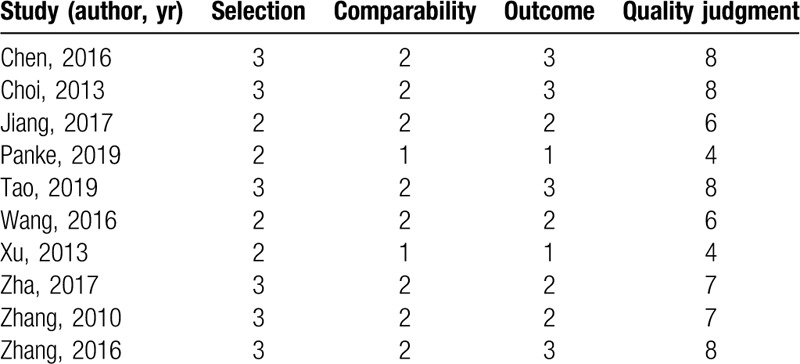
Quality assessment of studies in the meta-analysis based on Newcastle–Ottawa scale.

### 2.6 Data synthesis and statistical analysis

This study was statistical analyzed by using Review Manager Version 5.3.^[14]^ Most of included studies are retrospective cohort studies. So, odds ratio (OR) is used to calculate the dichotomous data in this meta-analysis. The continuous data were calculated by mean difference with 95% confidence interval (CI). We derived the missing standard deviations from other statistics, such as *P*-values or CI if needed. For example, *P* = .00001 was assumed when a *P*-value was reported as *P* < .00001. Cochran *Q* test and the degree of inconsistency (*I*^2^) were used to assess heterogeneity among combined study results. A fixed-effects model was used if a *P* > .05 and *I*^2^ < 50%. Otherwise, data were pooled by using the random-effects. *P* < .05 indicated statistical significance in the integration results. Publication bias in outcomes was assessed and treated using standard methodology. The funnel plots were used to analyze publication bias.

### 2.7 Study characteristics

**Figure 1:**
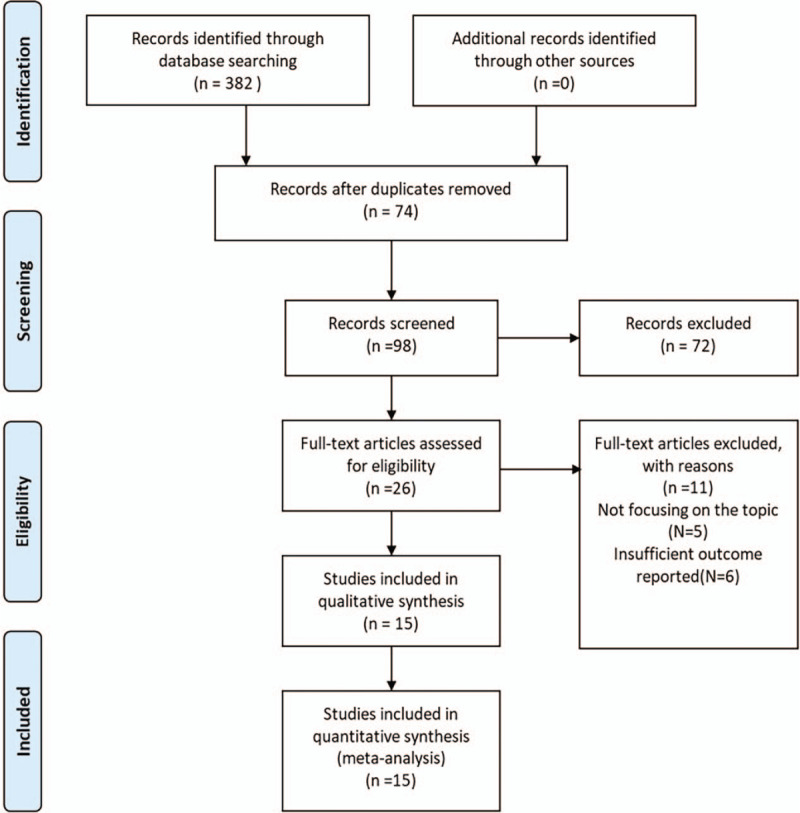
Flow diagram showing selection of relevant articles in the meta-analysis.

The detailed results of the search for relevant literature based on the strategy described above was shown in Figure 1. A total of 15 articles^[15–29]^ that enrolled 1156 patients (550 cases for PTED group and 606 cases for PIED group) met the inclusion criteria. All of the above literatures recruited Asians. Of the 15 studies, 5 articles^[17,19–21,23]^ were randomized studies, and 10 studies^[15,16,18,22,24–29]^ were retrospective studies. Of all study participants included had minimum 3-month follow-up. The concrete characteristics of the included studies were summarized in Table 3.

**Table 3:**
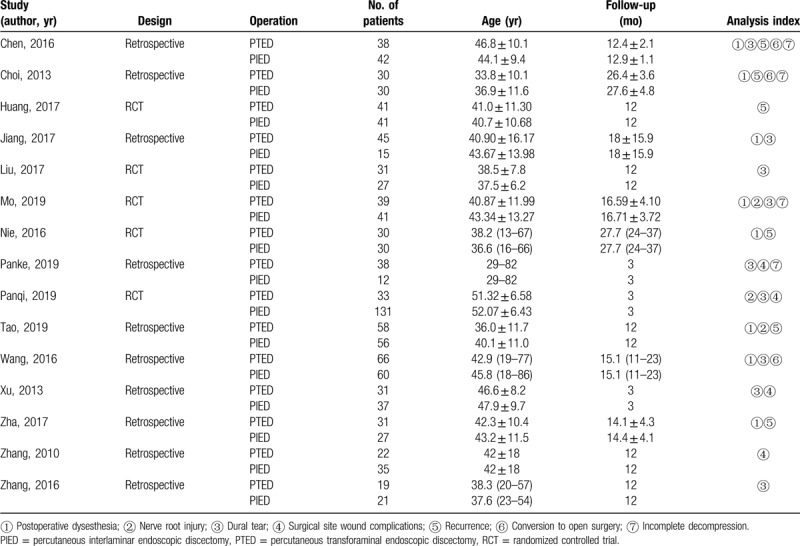
Baseline characteristics of the studies included in the meta-analysis.

### 2.8 Study quality assessment

According to the Cochrane Back Review Group (Table 1), The methodological quality score of 3 trials^[17,19,23]^ were 4 (high risk of bias), while the remaining 2 RCTs^[20,21]^ had quality scores of 7–8 (low risk of bias). The quality of nonrandomized trials was assessed by Newcastle–Ottawa scale (Table 2). Eight nonrandomized studies ranged from 5 to 8 points (low risk of bias) but 2 studies were 4 points (high risk of bias). In general, the quality of included studies was moderate to high.

## 3 Meta-analysis results

### 3.1 Postoperative dysesthesia

**Figure 2:**
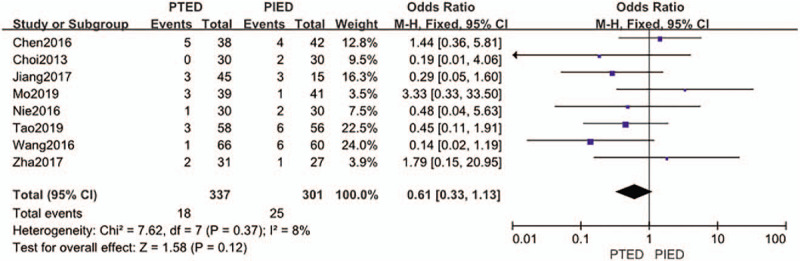
The forest plot for postoperative dysesthesia between PTED and PIED. PIED = percutaneous interlaminar endoscopic discectomy, PTED = percutaneous transforaminal endoscopic discectomy.

The postoperative dysesthesia was available from 8 studies.^[15,16,18,20,21,24,25,27]^ Postoperative dysesthesia occurred in 18 of 337 patients (5.3%) in PTED group, 25 of 301 patients (8.3%) in PIED group. Analysis indicated that there was low heterogeneity among the studies (*P* = .37, *I*^2^ = 8%) and a fixed effect model was used. Based on the complete analysis, postoperative dysesthesia did not differ significantly between PTED and PIED in the treatment of LDH (OR = 0.61, 95% CI, 0.33–1.13) (Fig. 2).

### 3.2 Nerve root injury

**Figure 3:**
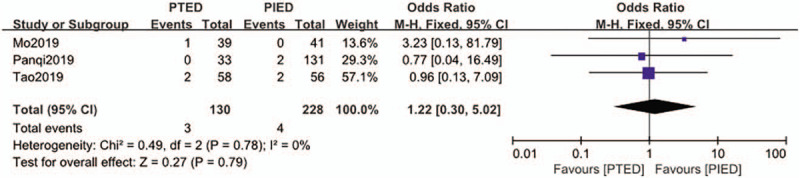
The forest plot for nerve root injury between PTED and PIED. PIED = percutaneous interlaminar endoscopic discectomy, PTED = percutaneous transforaminal endoscopic discectomy.

The nerve root injury was available from 3 studies.^[20,23,24]^ Nerve root injury showed in 3 of 130 patients (2.3%) in PTED group, 4 of 228 patients (1.8%) in PIED group. Analysis indicated that there was low heterogeneity among the studies (*P* = .78, *I*^2^ = 0%) and a fixed effect model was used. Based on the complete analysis, nerve root injury did not differ significantly between PTED and PIED in the treatment of LDH (OR = 1.22, 95% CI, 0.30–5.02) (Fig. 3).

### 3.3 Dural tear

**Figure 4:**
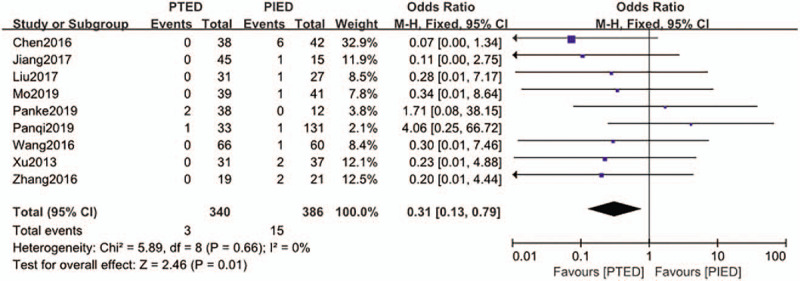
The forest plot for dural tear between PTED and PIED. PIED = percutaneous interlaminar endoscopic discectomy, PTED = percutaneous transforaminal endoscopic discectomy.

The dural tear was available from 9 studies.^[15,18–20,22,23,25,26,29]^ Dural tear occurred in 3 of 340 patients (0.9%) in PTED group, 15 of 386 patients (3.9%) in PIED group. Analysis indicated that there was low heterogeneity among the studies (*P* = .66, *I*^2^ = 0%) and a fixed effect model was used. Based on the complete analysis, dural tear was significantly less occured in PTED than in PIED (OR = 0.31, 95% CI, 0.13–0.79) (Fig. 4).

### 3.4 Surgical site wound complication

**Figure 5:**
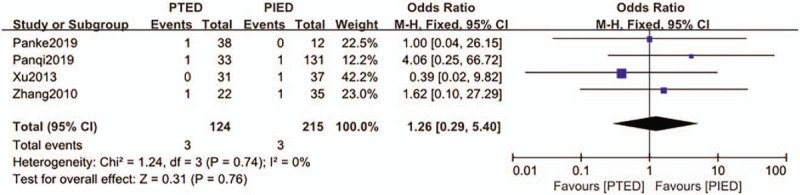
The forest plot for surgical site wound complications between PTED and PIED. PIED = percutaneous interlaminar endoscopic discectomy, PTED = percutaneous transforaminal endoscopic discectomy.

The surgical site wound complication was available from 4 studies.^[22,23,26,28]^ Surgical site wound complication occurred in 3 of 124 patients (2.4%) in PTED group, 3 of 215 patients (1.4%) in PIED group. Analysis indicated that there was low heterogeneity among the studies (*P* = .74, *I*^2^ = 0%) and a fixed effect model was used. Based on the complete analysis, surgical site wound complication did not differ significantly between PTED and PIED in the treatment of LDH (OR = 1.26, 95% CI, 0.29–5.40) (Fig. 5).

### 3.5 Recurrence

**Figure 6:**
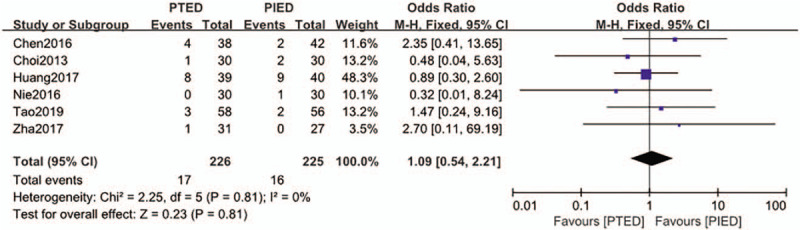
The forest plot for recurrence between PTED and PIED. PIED = percutaneous interlaminar endoscopic discectomy, PTED = percutaneous transforaminal endoscopic discectomy.

The recurrence was available from 6 studies.^[15–17,21,24,27]^ Recurrence occurred in 17 of 226 patients (7.5%) in PTED group, 16 of 225 patients (7.1%) in PIED group. Analysis indicated that there was low heterogeneity among the studies (*P* = .81, *I*^2^ = 0%) and a fixed effect model was used. Based on the complete analysis, recurrence did not differ significantly between PTED and PIED in the treatment of LDH (OR = 1.09, 95% CI, 0.54–2.21) (Fig. 6).

### 3.6 Conversion to open surgery

**Figure 7:**
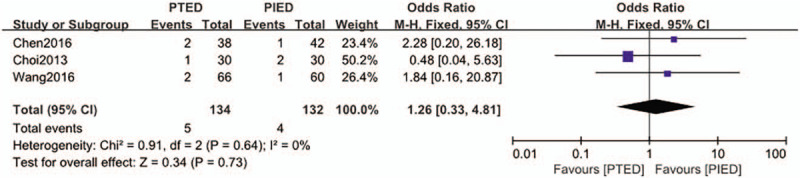
The forest plot for conversion to open surgery between PTED and PIED. PIED = percutaneous interlaminar endoscopic discectomy, PTED = percutaneous transforaminal endoscopic discectomy.

The conversion to open surgery was available from 3 studies.^[15,16,25]^ Conversion to open surgery found in 5 of 134 patients (3.7%) in PTED group, 4 of 132 patients (3.0%) in PIED group. Analysis indicated that there was low heterogeneity among the studies (*P* = .64, *I*^2^ = 0%) and a fixed effect model was used. Based on the complete analysis, conversion to open surgery did not differ significantly between PTED and PIED in the treatment of LDH (OR = 1.26, 95% CI, 0.33–4.81) (Fig. 7).

### 3.7 Incomplete decompression

**Figure 8:**
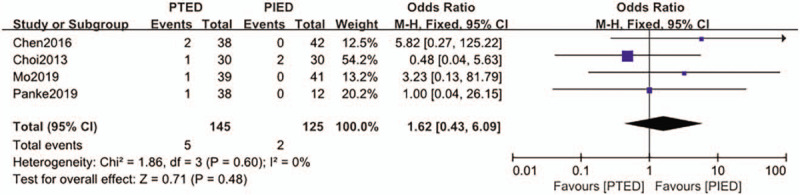
The forest plot for incomplete decompression between PTED and PIED. PIED = percutaneous interlaminar endoscopic discectomy, PTED = percutaneous transforaminal endoscopic discectomy.

The incomplete decompression was available from 4 studies.^[15,16,20,22]^ Incomplete decompression found in 5 of 145 patients (3.4%) in PTED group, 2 of 125 patients (1.6%) in PIED group. Analysis indicated that there was low heterogeneity among the studies (*P* = .60, *I*^2^ = 0%) and a fixed effect model was used. Based on the complete analysis, incomplete decompression did not differ significantly between PTED and PIED in the treatment of LDH (OR = 1.62, 95% CI, 0.43–6.09) (Fig. 8).

### 3.8 Total complication

**Figure 9:**
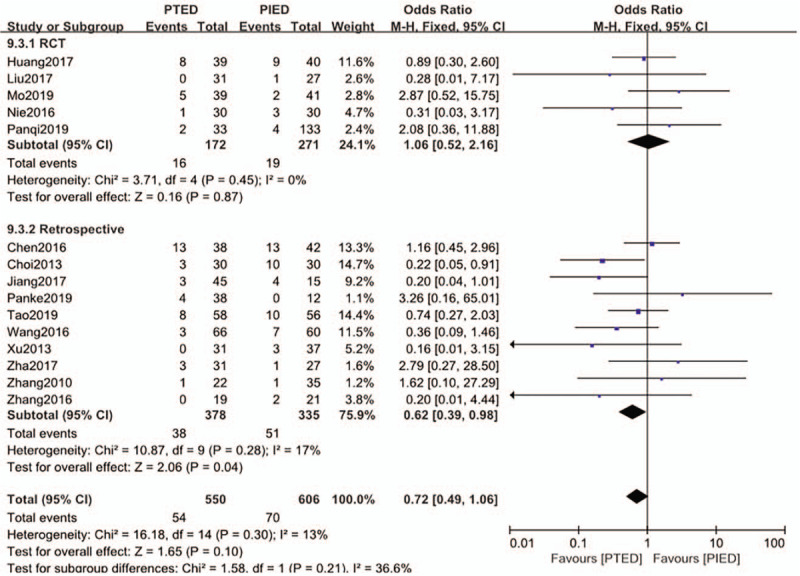
The forest plot for total complication between PTED and PIED. PIED = percutaneous interlaminar endoscopic discectomy, PTED = percutaneous transforaminal endoscopic discectomy.

The total complication was available from 15 studies.^[15–29]^ Total complications occurred in 54 of 550 patients (9.8%) in PTED group, 70 of 606 patients (11.6%) in PIED group. Analysis indicated that there was low heterogeneity among the studies (*P* = .30, *I*^2^ = 13%) and a fixed effect model was used. Based on the complete analysis, total complication did not differ significantly between PTED and PIED in the treatment of LDH (OR = 0.72, 95% CI, 0.49–1.06) (Fig. 9). Subgroup analysis evaluated just for retrospective studies showed significant difference (OR = 0.62, 95% CI, 0.39–0.98) in total complication between the 2 groups while 10 studies^[15,16,18,22,24–29]^ with a total of 713 patients included.

## 4 Discussion

In the PLED surgery, the structure of lumbar joint ligament was not damaged, and there was no significant effect on the stability of lumbar spine.^[30]^ What's more, during the procedure, there is no need to pull the nerve root and dural sac, no obvious disturbance to the nerve tissue in the spinal canal, no obvious bleeding and adhesion in the spinal canal, which has the advantages of small surgical trauma, short time in bed rest and quickly rehabilitation.^[5,31]^ PLED has been evolving from a state-of-the-art procedure into a more standard technique for treatment of extruded and/or migrated LDH, even stenosis. Although problems are unusual and infrequent, the potential complications associated with this procedure should be noted.^[10,32]^ This is the first meta-analysis to analyze the differences of complications between PTED and PIED in the treatment of LDH.

Nine (1.1%) of the 816 patients^[33]^ experienced symptomatic dural tears associated with PELD. Our data demonstrated that dural tear was occurred in 3 of 340 patients (0.9%) in PTED group, and 15 of 386 patients (3.9%) in PIED group. The treatment of paracentral and prolapsed subtype of LDH in PIED is relatively simple and easy to reach the target. The PIED approach utilizes the posterior IL approach, which matches the operation habits for most spine surgeons, has an easier identification of microscopic vision of the anatomic orientation than PTED. However, dealing with the central LDH in PIED often accompanied with difficulties and certain skills. It is often necessary to enter into the axil of the nerve root by increasing the inclination angle of the working cannula or rotating the working cannula, which increases the risk of dural tear and excessive traction of the nerve root. However, cannulation enters from the lateral intervertebral foramen without directly pulling the nerve root and dural sac in TF approach, which is not easy to lead to dural sac tear and excessive pulling of nerve root. What's more, with the change of the foraminoplasty tool from the trephine to the spiral bone drill, the probability of the dura and nerve root injury gradually decreased. Dural tears occurred in 26 of 835 patients (3.1%) in the study of Yorukoglu AG et al,^[34]^ and only 1 patient suffered a second surgery of dural repair after first time open microsurgical dural repair. Therefore, the author recommended not attempting dural repair if a dural tear occurs due to the limited access.

Sencer A et al reported^[35]^ that postoperative dysesthesias were encountered in 4 of 163 cases (2.4%), all of which were approached with a TF technique. Postoperative paresthesia had the highest incidence (15 cases, 3.1%) among all complications in Xie TH's^[36]^ observations following PIED. However, postoperative dysesthesia showed no significant difference between PTED and PIED in our article. Some studies^[30,37]^ have observed that intraoperative spinal ganglion or nerve root irritation may cause postoperative paresthesia. In addition, repeated puncture and overuse of bipolar radiofrequency scalpel are also the main causes of postoperative nerve root edema and neuritis. Therefore, the use of puncture and bipolar radiofrequency electrotome should be reduced during the operation, which may be able to avoid postoperative dysesthesia. Additionally, Xie TH et al^[36]^ found that the incidence of postoperative paresthesia among the patients with lateral recess stenosis (4 cases, 27%) was significantly greater than that among the patients without lateral recess stenosis. Aydn reported^[38]^ 11 cases of paresthesia in 857 patients that gradually improved following 2 to 6 weeks of rehabilitation and treatment with pregabalin. The symptoms often relieved after medical therapy, epidural, and foraminal steroid injections without any surgical interventions.

Nerve root injury^[39]^ is a common complication of both open microdiscectomy and PLED which incidence rate is 2.6% and 1.1%, respectively. We observed that it occurs in 3 of 130 patients (2.3%) in PTED group, 4 of 228 patients (1.8%) in PIED group. There are many reasons for nerve root injury during PLED via 2 approaches. First, the operation of PLED should be carried out under the condition of “2-dimensional” or even “blind vision” which cannot better distinguish the relationship of the nerve root with other tissues. Additionally, bleeding caused by an increase in blood pressure during the irritation of freeing the nerve root and inflammatory vasculature may blur the surgical field and affect the fluency and accuracy of the operation. Third, anatomical and radicular variations are common,^[40,41]^ especially in lumbosacral nerve. Lastly, excessive nerve root tension is also a factor of nerve root injury. Intrusion and extrusion of instruments in narrow epidural space may increase the intraspinal pressure and enhance the risk of nerve root injury. In some case of PIED, rotating the surgical cannula to push the nerve root to the midline to expose the protruding nucleus pulposus, which may increase its tension and shear the nerve root by the bevel on 1 side of the cannula. Unlike PIED, PTED is usually performed under local anesthesia, which improves the safety of the operation to a certain extent. However, no statistically significant differences were found between the 2 approaches based on our article. In order to avoid damaging the nerve root, the surgeon must know the depth of the device placement and be able to clearly identify the important anatomic structures around in a good surgical field. Furthermore, gentle and accurate manipulation in a step-by-step manner without anxiousness or roughness is required.

Surgical site wound complication includes superficial wound infection, deep intervertebral space infection, and low back pain caused by operation site. The total incidence of surgical site infection (SSI) after lumbar laminectomy and/or discectomy was 0.65% (26 cases; 17 deep SSI, 9 superficial SSI.^[42]^ We observed 6 patients suffered surgical site wound complication in 339 patients (1.8%), and only 1 patient was diagnosed as intervertebral infection (0.3%). There was 1 (0.79%) case of intervertebral space infection in 127 patients who underwent PIED in Liu Y's study.^[43]^ Additionally, In Yorukoglu AG's study^[34]^ of FELD, the infection rate was 0.14%, which was lower than the average infection rate of open surgery. The reason for this difference may be reduced hematoma in the operation field, reduced exposure of the operation field, and continuous irrigation of normal saline. Puncture needle entering intestine by mistake may cause the infection of intervertebral space while *Escherichia coli* was cultured from wound drainage fluid in a case in our hospital. In our experience, patients with mild symptoms and signs should be given antibiotics and bed rest. Patients with severe symptoms and signs should be treated with irrigation-suctioning in minimally invasive surgery firstly, and open surgical debridement should be done immediately when flushing and drainage are invalid.

Low back pain caused by operation site may be related to the muscle and soft tissue injury in puncture route, which can be improved gradually through rest. However, postoperative back pain which cannot be effectively relieved by rest and physical therapy has been linked to the biomechanics and the stability of lumbar spine. In order to effectively reduce the pressure and enlarge the working channel of endoscope, the bone structure of partial articular process is often excised to enlarge the intervertebral foramen in PTED. Qian Jun et al confirmed^[44]^ the biomechanics and the stability of lumbar spine changed partly after 1/4 resection of the superior articular process and obviously after more than 2/4 is resected. Although valuable clinical evidence has not yet been demonstrated, but superior articular process should be paid more attention during foraminotomy via PTED.

According to the result of meta-analysis of Si Yin et al,^[45]^ PELD is associated with a certain rate of recurrence (3.6%), the incidence of early recurrence was nearly double the late recurrence rate, and patients with early recurrence account for the majority of patients with recurrent herniations. And the prevalence estimates after PIED and PTED were 4.2% and 3.4%. In our study, the recurrence rate was 7.5% (17 of 226) in PTED group, and 7.1% (16 of 225) in PIED group respectively, which were higher than the average recurrence rate of study above. The reason for this difference may be fewer studies included, different follow-up times, and different proficiency in surgical techniques. Fewer cases in our included studies point out that there will be some insufficiencies in the early days of mastering a new technology. Kim et al^[46]^ suggest that annular sealing and annuloplasties can effectively reduce recurrence, particularly during the early stage. Some health education should be provided to the patients, such as the need for increased exercise, weight reduction, and the avoidance of smoking and drugs. Our included studies were mostly Chinese articles, the standard rehabilitation exercise after operation is always what we lack and need to improve. Furthermore, doctors should guide patients to perform back muscle exercises and suggest to their patients that they walk with the protection of a waist brace within the month following surgery.

Some scholars^[47]^ summarize 2 potential reasons for conversion from the IL approach to an open technique. First, misplacement of the working portal during the exposure of the ligament flavum can be problematic. Second, the herniation type can also have a great effect on the ease of this technique. In our experience, conversion to open surgery usually occurs in the process of puncture difficulty. Additionally, the patient cannot cooperate because of pain during the catheterization process, and the surgical field is unclear which surrounding anatomical structure cannot be distinguished may lead to switch to open surgery. With the improvement of surgical technique and proficiency, the difficulty of puncture can be overcome gradually. For the cases with narrow foramen and high iliac crest, especially in L5/S1, the IL approach can be used properly. However, for PTED patients with severe nerve root compression, the intubation process and arthroplasty may aggravate the compression and cause intense radiation pain under local anesthesia. The problem of prevention of bleeding and effective hemostasis have always been discussed by surgeons in PLED. Both the patients with large herniations and sundown type of nerve root as well as those patients with a longer duration of symptoms resulted in hyperplasia of small blood vessels, nerve root displacement, and adhesions surrounding the neural structures. This led to difficulties in dissecting the nerve root as well as hemostasis. The preoperative evaluation should be done well, and the safe working path, puncture point, puncture angle, and puncture depth should be determined according to the imaging data such as computed tomography or magnetic resonance imaging. In the key steps of X-ray real-time monitoring, the position of puncture needle, catheter and guide wire were detected. Hemostasis was strictly carried out in the operation area, and the extra dural fat and ligamentum flavum were preserved as much as possible to reduce the space of hematoma formation. Meanwhile, the hemoptysis in the operation area should be reduced by douching with fluid gelatin.

Safety aside, the adequacy of decompression has always been a concern of experienced surgeons in MISS. Lee et al^[48]^ indicated that the central-located high-canal compromised and high-grade migration herniations showed a high rate of incomplete decompression treated with PIED in 2006. However, in the study of Kim CH et al,^[49]^ complete removal of the highly migrated disc material by IL route was confirmed with MRI in 16 patients (success rate 89%) in 2016. Although some scholars^[50,51]^ reported successful surgery could be performed with the TF approach by an experienced hand, risk of root injury or remnant disk material is still present. The risk would increase with highly migrated disc due to the inclination of the endoscope in the neural foramen and the closeness of the exiting root with the endoscope. The scope of the manipulation of the working cannula in PIED is considerably larger than that in PTED; Thus, we give priority to the use of PIED in the treatment of migratory herniations. Incomplete decompression is related to preoperative diagnosis error, intraoperative negligence, and lack of experience of surgeons. The location and size of the nucleus pulposus protrusion should be determined in accordance with the preoperative imaging, especially MRI within 1 week.^[52]^ However, intraoperative observation and assessment is often accepted by surgeons to ensure the end-point of the procedure. When the nerve was sufficiently decompressed, a favorable mobility of the nerve root could be observed by adjusting the hydraulic pressure by repeatedly opening and closing the hydro valve of the endoscope both in PTED and PIED. Moreover, it is important to examine the residual fragments in the shoulder, axillary areas, and track of the nerve root carefully before the end-point is reached. Because of the difficult learning curve and the lack of skill with requisite surgical techniques, different kinds of problems can arise with some frequency, especially when complications cause severe injury for the patient. Surgeons should vigilantly make adequate technical considerations and thoroughly understand patient anatomy to avoid complications.

In this study, there was no significant difference between the total complications of the 2 surgical techniques. However, due to the mild heterogeneity, a subgroup analysis was carried out, and the results showed that the total complication rate of the PTED in the retrospective study was significantly lower than that of the PIED. In the randomized control study, there was no significant difference between the total complications of PTED and PIED. The reason may be that there is a large bias in the observational study, which leads to a large heterogeneity in the study.

## 5 Conclusions

Dural tear was significantly less occured in PTED compared with PIED. The postoperative dysesthesia, nerve root injury, surgical site wound complications, recurrence, conversion to open surgery, incomplete decompression, reoperations, and total complication did not differ significantly between PTED and PIED in the treatment of LDH. High-quality RCTs with sufficiently larger sample sizes and longer follow-up period are necessary to further confirm these results.

## Author contributions

**Conceptualization:** Luming Nong.

**Data curation:** Jianjian Yin, Yuqing Jiang.

**Formal analysis:** Jianjian Yin, Yuqing Jiang.

**Investigation:** Jianjian Yin, Yuqing Jiang.

**Methodology:** Jianjian Yin, Yuqing Jiang.

**Software:** Jianjian Yin.

**Supervision:** Jianjian Yin, Luming Nong.

**Writing – original draft:** Jianjian Yin, Yuqing Jiang.

**Writing – review & editing:** Luming Nong.

## References

[1] Mixter WJ Barr JS Rupture of the intervertebral disc with involvement of the spinal canal. New Engl J Med 1934;211:210–5.

[2] Arts M Brand R van der Kallen B Does minimally invasive lumbar disc surgery result in less muscle injury than conventional surgery? A randomized controlled trial. Eur Spine J 2011;20:51–7. 20556439 10.1007/s00586-010-1482-y PMC3036021

[3] Zhou C Zhang G Panchal RR Unique complications of percutaneous endoscopic lumbar discectomy and percutaneous endoscopic interlaminar discectomy. Pain Physician 2018;21:E105–12. 29565953

[4] Gadjradj PS van Tulder MW Dirven CM Clinical outcomes after percutaneous transforaminal endoscopic discectomy for lumbar disc herniation: a prospective case series. Neurosurg Focus 2016;40:E3. 10.3171/2015.10.FOCUS15484 26828884

[5] Li X Han Y Di Z Percutaneous endoscopic lumbar discectomy for lumbar disc herniation. J Clin Neurosci 2016;33:19–27. 27475315 10.1016/j.jocn.2016.01.043

[6] Li ZZ Hou SX Shang WL The strategy and early clinical outcome of full-endoscopic L5/S1 discectomy through interlaminar approach. Clin Neurol Neurosurg 2015;133:40–5. 25837573 10.1016/j.clineuro.2015.03.003

[7] Komp MG Ruetten GS Bilateral spinal decompression of lumbar central stenosis with the full-endoscopic interlaminar versus microsurgical laminotomy technique: a prospective, randomized, controlled study. Pain Physician 2015;18:61–70. 25675060

[8] Yeung AT Tsou PM Posterolateral endoscopic excision for lumbar disc herniation: surgical technique, outcome, and complications in 307 consecutive cases. Spine (Phila Pa 1976) 2002;27:722–31. 11923665 10.1097/00007632-200204010-00009

[9] Hsu HT Chang SJ Yang SS Learning curve of full-endoscopic lumbar discectomy. Eur Spine J 2013;22:727–33. 23076645 10.1007/s00586-012-2540-4 PMC3631049

[10] Sairyo K Matsuura T Higashino K Surgery related complications in percutaneous endoscopic lumbar discectomy under local anesthesia. J Med Invest 2014;61:264–9. 25264043 10.2152/jmi.61.264

[11] Moher D Liberati A Tetzlaff J Preferred reporting items for systematic reviews and meta-analyses: the PRISMA statement. PLoS Med 2009;6:e1000097. 19621072 10.1371/journal.pmed.1000097 PMC2707599

[12] Furlan AD Pennick V Bombardier C Editorial board CBRG 2009 updated method guidelines for systematic reviews in the Cochrane Back Review Group. Spine 2009;34:1929–41. 19680101 10.1097/BRS.0b013e3181b1c99f

[13] Stang A Critical evaluation of the Newcastle-Ottawa scale for the assessment of the quality of nonrandomized studies in meta-analyses. Eur J Epidemiol 2010;25:603–5. 20652370 10.1007/s10654-010-9491-z

[14] Reeves BC Higgins JP Ramsay C An introduction to methodological issues when including non-randomised studies in systematic reviews on the effects of interventions [published correction appears in Res Synth Methods. 2013 Sep;4(3):287-9]. *Res Synth Methods*. 2013;4:1-11. doi:10.1002/jrsm.1068 10.1002/jrsm.1068 26053535

[15] Chen CQ Wang C Wang YS The comparison of two kinds of percutaneous endoscopic approach for treatment of L5/S1 disc herniation. Chin J Trad Med Traum Orthop 2016;24:25–8.

[16] Choi KC Kim JS Ryu KS Percutaneous endoscopic lumbar discectomy for L5-S1 disc herniation: transforaminal versus interlaminar approach. Pain Physician 2013;16:547–56. 24284840

[17] Huang H Yang B Song JT The comparison of therapeutic effects between two different approaches in percutaneous endoscopic lumbar discectomy for L5S1 lumbar disc herniation. Lab Med Clin 2017;14:1651–3.

[18] Jiang Y Zuo R-J Wu L Surgical outcome of percutaneous endoscopic technique for highly migrated disc herniation via three different approaches. China J Orthop Traumatol 2017;30:100–4. 10.3969/j.issn.1003-0034.2017.02.002 29349997

[19] Liu C Chu L Yong HC Percutaneous endoscopic lumbar discectomy for highly migrated lumbar disc herniation. Pain Physician 2017;20:E75–84. 28072799

[20] Mo X Shen J Jiang W Percutaneous endoscopic lumbar diskectomy for axillar herniation at L5-S1 via the transforaminal approach versus the interlaminar approach: a prospective clinical trial. World Neurosurg 2019;125:e508–14. 30710722 10.1016/j.wneu.2019.01.114

[21] Nie H Zeng J Song Y Percutaneous endoscopic lumbar discectomy for L5-S1 disc herniation via an interlaminar approach versus a transforaminal approach: a prospective randomized controlled study with 2-year follow up. Spine (Phila Pa 1976) 2016;41: Suppl 19: B30–7. 27454540 10.1097/BRS.0000000000001810

[22] Pan K Li Z Peng H Clinical analysis of 50 cases of lumbar disc herniation treated with percutaneous lumbar endoscopic discectomy via transforaminal approach and translaminar approach. China Prac Med 2019;14:13–5.

[23] Pan Q Wang L Wu Y Effect of endoscopic surgery by interlaminar and transforaminal approach in the treatment of lumbar intervertebral disc protrusion. J Xinxiang Med Univ 2019;36:1060–3.

[24] Tao H Zhang Y Dong F Comparison of the clinical effects of interlaminar and transforaminal percutaneous endoscopic discectomy for L5-S1 disc herniation. J Cervicodynia Lumbodynia 2019;40:587–94.

[25] Wang Z Kan F Wang X A comparison of the transforaminal versus the interlaminar approach to percutaneous endoscopic surgeries in treating lumbar disc herniation. Chin J Neurosurg 2016;32:1214–9.

[26] Xu Z Shi L Chu L Comparison of percutaneous endoscopic via interlaminar and transforaminal approach for lumbar disc herniation. J Spinal Sur 2013;11:97–100.

[27] Zha YY Jin W Zhang SW Clinical effects of percutaneous endoscopic L5/S1 lumbar discectomy through transforaminal approach and interlaminar approach. Orthop Biomech Mater Clin Study 2017;14:64–8.

[28] Zhang X Wang Y Xiao S Treatment of L5/S1 disc herniation under endoscope via postlateral transforaminal and interlaminar approach. Chin J Orthop 2010;30:341–5.

[29] Zhang JH Chen DX Zhang JX Clinical observation of curative effect of TESSYS and PEID for treatment of L5/S1 disc herniation. Chin J Bone Joint Inj 2016;31:929–32.

[30] Ruetten S Komp M Merk H Full-endoscopic interlaminar and transforaminal lumbar discectomy versus conventional microsurgical technique: a prospective, randomized, controlled study. Spine 2008;33:931–9. 18427312 10.1097/BRS.0b013e31816c8af7

[31] Rasouli MR Rahimi-Movaghar V Shokraneh F Minimally invasive discectomy versus microdiscectomy/open discectomy for symptomatic lumbar disc herniation. Cochrane Database Syst Rev 2014;CD010328 Published 2014 Sep 4. doi:10.1002/14651858.CD010328.pub2. 25184502 10.1002/14651858.CD010328.pub2 PMC10961733

[32] Xi T-H Zeng J-C Li Z-H Complications of lumbar disc herniation following full-endoscopic interlaminar lumbar discectomy: a large, single-center, retrospective study. Pain Physician 2017;20:E379–87. 28339437

[33] Ahn Y Lee HY Lee SH Dural tears in percutaneous endoscopic lumbar discectomy. Eur Spine J 2011;20:58–64. 20582555 10.1007/s00586-010-1493-8 PMC3036033

[34] Yörükoğlu AG Göker B Tahta A Fully endoscopic interlaminar and transforaminal lumbar discectomy: analysis of 47 complications encountered in a series of 835 patients. Neurocirugia (Astur) 2017;28:235–41. 28532963 10.1016/j.neucir.2017.03.003

[35] Sencer A Yorukoglu AG Akcakaya MO Fully endoscopic interlaminar and transforaminal lumbar discectomy: short-term clinical results of 163 surgically treated patients. World Neurosurg 2014;82:884–90. 24907438 10.1016/j.wneu.2014.05.032

[36] Xie TH Zeng JC Li ZH Complications of lumbar disc herniation following full-endoscopic interlaminar lumbar discectomy: a large, single-center, retrospective study. Pain Physician 2017;20:E379–87. 28339437

[37] Cho JY Lee SH Lee HY Prevention of development of postoperative dysesthesia in transforaminal percutaneous endoscopic lumbar discectomy for intracanalicular lumbar disc herniation: floating retraction technique. Minim Invasive Neurosurg 2011;54:214–8. 22287030 10.1055/s-0031-1287774

[38] Aydn S Bolat E Fully endoscopic interlaminar and transforaminal lumbar discectomy: clinical results of 857 surgically treated patients. Neurol Neurochir Pol 2019;53:492–9. 31804701 10.5603/PJNNS.a2019.0064

[39] Shriver MF Xie JJ Tye EY Lumbar microdiscectomy complication rates: a systematic review and meta-analysis. Neurosurg Focus 2015;39:E6 doi:10.3171/2015.7. FOCUS 15281. 10.3171/2015.7.FOCUS15281 26424346

[40] Postacchini F Urso S Ferro L Lumbosacral nerve-root anomalies. J Bone Joint Surg Am 1982;64:721–9. 7085697

[41] Chotigavanich C Sawangnatra S Anomalies of the lumbosacral nerve roots. An anatomic investigation. Clin Orthop Relat Res 1992;46–50. 1563168

[42] Ogihara S Yamazaki T Inanami H Risk factors for surgical site infection after lumbar laminectomy and/or discectomy for degenerative diseases in adults: a prospective multicenter surveillance study with registry of 4027 cases. PLoS One 2018;13:e0205539. 30325940 10.1371/journal.pone.0205539 PMC6191117

[43] Liu Y Wang S Yang C Retrospective study of the interlaminar approach for percutaneous endoscopic lumbar discectomy with the guidance of pre-operative magnetic resonance neurography. Ann Transl Med 2019;7:145. 31157266 10.21037/atm.2019.03.22 PMC6511578

[44] Jun Q Shuisheng Y Jianjun L Biomechanics changes of lumbar spine caused by foraminotomy via percutaneous transforaminal endoscopic lumbar discectomy. Natl Med J China 2018;98:1013–8. 10.3760/cma.j.issn.0376-2491.2018.13.012 29690712

[45] Yin S Du H Yang W Prevalence of recurrent herniation following percutaneous endoscopic lumbar discectomy: a meta-analysis. Pain Physician 2018;21:337–50. 30045591

[46] Kim HS Park JY Comparative assessment of different percutaneous endoscopic interlaminar lumbar discectomy (PEID) techniques. Pain Physician 2013;16:359–67. 23877452

[47] Wang B Lü G Liu W Full-endoscopic interlaminar approach for the surgical treatment of lumbar disc herniation: the causes and prophylaxis of conversion to open. Arch Orthop Trauma Surg 2012;132:1531–8. 22763864 10.1007/s00402-012-1581-9

[48] Lee SH Kang BU Ahn Y Operative failure of percutaneous endoscopic lumbar discectomy: a radiologic analysis of 55 cases. Spine (Phila Pa 1976) 2006;31:E285–90. 16648734

[49] Kim CH Chung CK Woo JW Surgical outcome of percutaneous endoscopic interlaminar lumbar discectomy for highly migrated disk herniation. Clin Spine Surg 2016;29:E259–66. 23073149 10.1097/BSD.0b013e31827649ea

[50] Kim HS Ju CI Kim SW Endoscopic transforaminal suprapedicular approach in high grade inferior migrated lumbar disc herniation. J Korean Neurosurg Soc 2009;45:67–73. 19274114 10.3340/jkns.2009.45.2.67 PMC2651558

[51] Choi G Lee SH Lokhande P Percutaneous endoscopic approach for highly migrated intracanal disc herniations by foraminoplastic technique using rigid working channel endoscope. Spine (Phila Pa 1976) 2008;33:E508–15. 18594449 10.1097/BRS.0b013e31817bfa1a

[52] Ahn Y Transforaminal percutaneous endoscopic lumbar discectomy: technical tips to prevent complications. Expert Rev Med Devices 2012;9:361–6. 22905840 10.1586/erd.12.23

